# Supplementary material for: Combined Proxies for Heart Rate Variability as a Global Tool to Assess and Monitor Autonomic Dysregulation in Fibromyalgia and Disease-Related Impairments
Source: Sensors (Basel). 2025 Apr 21;25(8):2618. doi: 10.3390/s25082618 (PMC12031131; doi:10.3390/s25082618)
Supplement: Supplementary file 1 [file sensors-25-02618-s001.zip › sensors-3553002-supplementary.pdf]

Multivariate analyses controlled for age

Correlations

	VLF rs	LF rs	HF	Total Power	LF/HF rs	SD1 rs	SD2 rs	Corr.Dim. D2 rs	mean RR rs	SDNN rs	mean HR rs	RMSSD rs	NN50 rs	pNN50 rs	RR tri index rs	TINN rs	Illness duration	SAS	SDS	MAF	MOS	FIQ	NRS	SF-36_PHI	SF-36_MHI	VPI	SS	HRV grade
VLF rs	1,0000	0,6084	0,4594	0,8867	0,4125	0,2949	0,7567	0,4903	0,3531	0,6028	-0,3327	0,4160	0,4524	0,4939	0,7346	0,1168	0,0404	0,2558	-0,0436	0,0216	-0,0380	-0,1151	0,0287	0,0301	-0,1308	-0,2359	-0,1082	-0,4766
LF rs	0,6084	1,0000	0,6598	0,6468	0,2331	0,3704	0,7500	0,5991	0,0988	0,7017	-0,1003	0,5082	0,6629	0,5772	0,7181	0,3969	0,2529	0,3230	0,0656	0,1169	0,0837	-0,2455	0,0606	0,0736	-0,1629	-0,1430	0,0359	-0,5219
HF	0,4594	0,6598	1,0000	0,6683	0,0357	0,6567	0,7536	0,5705	0,2788	0,8095	-0,2222	0,8496	0,7182	0,7032	0,7026	0,4255	0,3246	0,2248	0,0595	0,0751	-0,0027	-0,3097	-0,0640	0,1304	-0,2192	-0,0497	0,1899	-0,6219
Total Power	0,8867	0,6468	0,6683	1,0000	0,3556	0,4444	0,8021	0,5702	0,3243	0,7301	-0,2942	0,6079	0,5416	0,5688	0,7217	0,2855	0,0357	0,3088	-0,0030	0,0737	-0,0007	-0,1424	0,0045	0,0858	-0,1783	-0,1999	-0,0230	-0,6248
LF/HF rs	0,4125	0,2331	0,0357	0,3556	1,0000	0,0195	0,1575	0,1681	0,0073	0,1379	-0,0216	0,0368	0,1551	0,1224	0,2687	0,0583	-0,0100	0,0274	-0,0227	0,1390	-0,0260	0,0154	-0,0143	-0,1891	-0,1132	-0,3445	-0,3447	-0,1634
SD1 rs	0,2949	0,3704	0,6567	0,4444	0,0195	1,0000	0,5536	0,2921	0,3601	0,6297	-0,3063	0,7741	0,4066	0,4081	0,3761	0,3197	0,3552	-0,1248	-0,1583	0,0203	0,1344	-0,2962	-0,0073	0,2240	0,0414	-0,0497	0,1012	-0,4180
SD2 rs	0,7567	0,7500	0,7536	0,8021	0,1575	0,5536	1,0000	0,7014	0,3772	0,9447	-0,3508	0,7799	0,6555	0,6666	0,9231	0,4391	0,1977	0,3388	-0,0408	-0,0329	0,0937	-0,3428	0,0115	0,1258	-0,0746	-0,1524	0,0294	-0,6884
Corr.Dim. D2 rs	0,4903	0,5991	0,5705	0,5702	0,1681	0,2921	0,7014	1,0000	0,3757	0,6213	-0,3506	0,4517	0,7619	0,7363	0,8420	0,2410	0,0591	0,2916	-0,0319	-0,0991	0,0383	-0,4567	-0,0572	0,1766	-0,0275	-0,0320	-0,0304	-0,5628
mean RR rs	0,3531	0,0988	0,2788	0,3243	0,0073	0,3601	0,3772	0,3757	1,0000	0,3532	-0,9419	0,3154	0,3846	0,5055	0,4150	-0,2220	-0,0588	-0,0442	-0,2051	-0,1351	-0,0154	-0,3981	-0,2617	0,3469	0,1889	0,1086	0,1026	-0,3993
SDNN rs	0,6028	0,7017	0,8095	0,7301	0,1379	0,6297	0,9447	0,6213	0,3532	1,0000	-0,3197	0,8624	0,6250	0,6423	0,8403	0,4763	0,2539	0,2593	-0,0384	0,0653	0,0738	-0,3510	0,0286	0,0786	-0,0577	-0,1185	0,0738	-0,6877
mean HR rs	-0,3327	-0,1003	-0,2222	-0,2942	-0,0216	-0,3063	-0,3508	-0,3506	-0,9419	-0,3197	1,0000	-0,2567	-0,3135	-0,4071	-0,4009	0,1385	0,0452	0,0586	0,2638	0,1730	0,0504	0,3777	0,2622	-0,3409	-0,2211	-0,0667	-0,0346	0,3933
RMSSD rs	0,4160	0,5082	0,8496	0,6079	0,0368	0,7741	0,7799	0,4517	0,3154	0,8624	-0,2567	1,0000	0,5522	0,5496	0,6601	0,5354	0,3553	0,1209	0,0210	-0,0002	0,0917	-0,3243	-0,0935	0,0961	-0,1279	-0,0663	0,1379	-0,6212
NN50 rs	0,4524	0,6629	0,7182	0,5416	0,1551	0,4066	0,6555	0,7619	0,3846	0,6250	-0,3135	0,5522	1,0000	0,9663	0,7385	0,1232	0,1756	0,2256	-0,0115	-0,0587	-0,0387	-0,3957	-0,0124	0,1800	-0,0400	0,0071	0,1014	-0,5628
pNN50 rs	0,4939	0,5772	0,7032	0,5688	0,1224	0,4081	0,6666	0,7363	0,5055	0,6423	-0,4071	0,5496	0,9663	1,0000	0,7387	0,0165	0,1160	0,2295	-0,0455	-0,0386	-0,0838	-0,3806	0,0234	0,1795	0,0083	0,0281	0,1080	-0,5419
RR tri index rs	0,7346	0,7181	0,7026	0,7217	0,2687	0,3761	0,9231	0,8420	0,4150	0,8403	-0,4009	0,6601	0,7385	0,7387	1,0000	0,2700	0,1467	0,3709	-0,0389	-0,0499	-0,0036	-0,3976	-0,0133	0,1591	-0,0906	-0,0919	0,0035	-0,6185
TINN rs	0,1168	0,3969	0,4255	0,2855	0,0583	0,3197	0,4391	0,2410	-0,2220	0,4763	0,1385	0,5354	0,1232	0,0165	0,2700	1,0000	0,1495	0,0537	0,0637	-0,1416	0,1684	-0,1283	-0,1281	-0,0486	-0,1148	-0,1003	-0,0354	-0,4441
Illness duration	0,0404	0,2529	0,3246	0,0357	-0,0100	0,3552	0,1977	0,0591	-0,0588	0,2539	0,0452	0,3553	0,1756	0,1160	0,1467	0,1495	1,0000	0,0881	0,0023	0,1974	-0,1831	-0,1564	0,1830	-0,1395	-0,1827	0,2070	0,2082	-0,0672
SAS	0,2558	0,3230	0,2248	0,3088	0,0274	-0,1248	0,3388	0,2916	-0,0442	0,2593	0,0586	0,1209	0,2256	0,2295	0,3709	0,0537	0,0881	1,0000	0,5969	0,2220	-0,2138	0,2959	0,2206	-0,0601	-0,6406	-0,0565	0,2932	0,0265
SDS	-0,0436	0,0656	0,0595	-0,0030	-0,0227	-0,1583	-0,0408	-0,0319	-0,2051	-0,0384	0,2638	0,0210	-0,0115	-0,0455	-0,0389	0,0637	0,0023	0,5969	1,0000	0,4439	-0,1131	0,4773	0,1818	-0,4441	-0,7694	0,1415	0,3869	0,1608
MAF	0,0216	0,1169	0,0751	0,0737	0,1390	0,0203	-0,0329	-0,0991	-0,1351	0,0653	0,1730	-0,0002	-0,0587	-0,0386	-0,0499	-0,1416	0,1974	0,2220	0,4439	1,0000	-0,1287	0,4900	0,3423	-0,5048	-0,3450	0,1580	0,2124	0,2596
MOS	-0,0380	0,0837	-0,0027	-0,0007	-0,0260	0,1344	0,0937	0,0383	-0,0154	0,0738	0,0504	0,0917	-0,0387	-0,0838	-0,0036	0,1684	-0,1831	-0,2138	-0,1131	-0,1287	1,0000	-0,2921	-0,2686	0,1849	0,0543	-0,1619	-0,0646	-0,1160
FIQ	-0,1151	-0,2455	-0,3097	-0,1424	0,0154	-0,2962	-0,3428	-0,4567	-0,3981	-0,3510	0,3777	-0,3243	-0,3957	-0,3806	-0,3976	-0,1283	-0,1564	0,2959	0,4773	0,4900	-0,2921	1,0000	0,6976	-0,5720	-0,4171	-0,0970	0,0475	0,5058
NRS	0,0287	0,0606	-0,0640	0,0045	-0,0143	-0,0073	0,0115	-0,0572	-0,2617	0,0286	0,2622	-0,0935	-0,0124	0,0234	-0,0133	-0,1281	0,1830	0,2206	0,1818	0,3423	-0,2686	0,6976	1,0000	-0,2867	-0,0621	-0,0762	0,0743	0,2799
SF-36_PHI	0,0301	0,0736	0,1304	0,0858	-0,1891	0,2240	0,1258	0,1766	0,3469	0,0786	-0,3409	0,0961	0,1800	0,1795	0,1591	-0,0486	-0,1395	-0,0601	-0,4441	-0,5048	0,1849	-0,5720	-0,2867	1,0000	0,2597	-0,1012	-0,1233	-0,1762
SF-36_MHI	-0,1308	-0,1629	-0,2192	-0,1783	-0,1132	0,0414	-0,0746	-0,0275	0,1889	-0,0577	-0,2211	-0,1279	-0,0400	0,0083	-0,0906	-0,1148	-0,1827	-0,6406	-0,7694	-0,3450	0,0543	-0,4171	-0,0621	0,2597	1,0000	0,0656	-0,2531	-0,0607
VPI	-0,2359	-0,1430	-0,0497	-0,1999	-0,3445	-0,0497	-0,1524	-0,0320	0,1086	-0,1185	-0,0667	-0,0663	0,0071	0,0281	-0,0919	-0,1003	0,2070	-0,0565	0,1415	0,1580	-0,1619	-0,0970	-0,0762	-0,1012	0,0656	1,0000	0,6501	0,1223
SS	-0,1082	0,0359	0,1899	-0,0230	-0,3447	0,1012	0,0294	-0,0304	0,1026	0,0738	-0,0346	0,1379	0,1014	0,1080	0,0035	-0,0354	0,2082	0,2932	0,3869	0,2124	-0,0646	0,0475	0,0743	-0,1233	-0,2531	0,6501	1,0000	0,0415
HRV grade	-0,4766	-0,5219	-0,6219	-0,6248	-0,1634	-0,4180	-0,6884	-0,5628	-0,3993	-0,6877	0,3933	-0,6212	-0,5628	-0,5419	-0,6185	-0,4441	-0,0672	0,0265	0,1608	0,2596	-0,1160	0,5058	0,2799	-0,1762	-0,0607	0,1223	0,0415	1,0000

Correlation probability

	VLF rs	LF rs	HF	Total Power	LF/HF rs	SD1 rs	SD2 rs	Corr.Dim. D2 rs	mean RR rs	SDNN rs	mean HR rs	RMSSD rs	NN50 rs	pNN50 rs	RR tri index rs	TINN rs	Illness duration	SAS	SDS	MAF	MOS	FIQ	NRS	SF-36_PHI	SF-36_MHI	VPI	SS	HRV grade
VLF rs	<,0001	<,0001	<,0001	<,0001	<,0001	0,0065	<,0001	<,0001	0,0010	<,0001	0,0020	<,0001	<,0001	<,0001	<,0001	0,2902	0,7572	0,0619	0,7521	0,8759	0,7828	0,2999	0,8350	0,8340	0,3603	0,0352	0,3392	<,0001
LF rs	<,0001	<,0001	<,0001	<,0001	0,0328	0,0005	<,0001	<,0001	0,3713	<,0001	0,3639	<,0001	<,0001	<,0001	<,0001	0,0002	0,0493	0,0172	0,6340	0,3955	0,5434	0,0253	0,6605	0,6077	0,2534	0,2059	0,7520	<,0001
HF	<,0001	<,0001	<,0001	<,0001	0,7473	<,0001	<,0001	<,0001	0,0102	<,0001	0,0422	<,0001	<,0001	<,0001	<,0001	<,0001	0,0107	0,1023	0,6658	0,5857	0,9846	0,0044	0,6425	0,3617	0,1223	0,6613	0,0916	<,0001
Total Power	<,0001	<,0001	<,0001	<,0001	0,0010	<,0001	<,0001	<,0001	0,0028	<,0001	0,0069	<,0001	<,0001	<,0001	<,0001	0,0089	0,7846	0,0244	0,9827	0,5966	0,9959	0,2019	0,9741	0,5537	0,2155	0,0774	0,8407	<,0001
LF/HF rs	<,0001	0,0328	0,7473	0,0010	<,0001	0,8604	0,1525	0,1264	0,9471	0,2111	0,8453	0,7411	0,1590	0,2673	0,0140	0,5983	0,9389	0,8441	0,8692	0,3116	0,8507	0,8904	0,9174	0,1839	0,4291	0,0018	0,0017	0,1375
SD1 rs	0,0065	0,0005	<,0001	<,0001	0,8604	<,0001	<,0001	0,0070	0,0008	<,0001	0,0046	<,0001	0,0001	0,0001	0,0005	0,0030	0,0050	0,3686	0,2484	0,8829	0,3280	0,0066	0,9579	0,1140	0,7729	0,6616	0,3716	<,0001
SD2 rs	<,0001	<,0001	<,0001	<,0001	0,1525	<,0001	<,0001	<,0001	0,0004	<,0001	0,0011	<,0001	<,0001	<,0001	<,0001	<,0001	0,1266	0,0122	0,7675	0,8116	0,4963	0,0015	0,9338	0,3789	0,6030	0,1770	0,7959	<,0001
Corr.Dim. D2 rs	<,0001	<,0001	<,0001	<,0001	0,1264	0,0070	<,0001	<,0001	0,0004	<,0001	0,0011	<,0001	<,0001	<,0001	<,0001	0,0272	0,6509	0,0324	0,8170	0,4718	0,7815	<,0001	0,6783	0,2151	0,8482	0,7781	0,7891	<,0001
mean RR rs	0,0010	0,3713	0,0102	0,0028	0,9471	0,0008	0,0004	0,0004	<,0001	0,0010	<,0001	0,0037	0,0003	<,0001	<,0001	0,0424	0,6525	0,7512	0,1330	0,3256	0,9113	0,0002	0,0536	0,0126	0,1844	0,3375	0,3653	0,0002
SDNN rs	<,0001	<,0001	<,0001	<,0001	0,2111	<,0001	<,0001	<,0001	0,0010	<,0001	0,0030	<,0001	<,0001	<,0001	<,0001	<,0001	0,0483	0,0584	0,7810	0,6356	0,5925	0,0011	0,8360	0,5836	0,6873	0,2953	0,5153	<,0001
mean HR rs	0,0020	0,3639	0,0422	0,0069	0,8453	0,0046	0,0011	0,0011	<,0001	0,0030	<,0001	0,0192	0,0037	0,0001	0,0002	0,2090	0,7292	0,6736	0,0516	0,2065	0,7148	0,0004	0,0532	0,0144	0,1189	0,5567	0,7607	0,0002
RMSSD rs	<,0001	<,0001	<,0001	<,0001	0,7411	<,0001	<,0001	<,0001	0,0037	<,0001	0,0192	<,0001	<,0001	<,0001	<,0001	<,0001	0,0050	0,3884	0,8804	0,9988	0,5097	0,0030	0,5013	0,5069	0,3760	0,5614	0,2257	<,0001
NN50 rs	<,0001	<,0001	<,0001	<,0001	0,1590	0,0001	<,0001	<,0001	0,0003	<,0001	0,0037	<,0001	<,0001	<,0001	<,0001	0,2640	0,1758	0,1010	0,9337	0,6704	0,7793	0,0002	0,9285	0,2062	0,7803	0,9503	0,3708	<,0001
pNN50 rs	<,0001	<,0001	<,0001	<,0001	0,2673	0,0001	<,0001	<,0001	<,0001	<,0001	0,0001	<,0001	<,0001	<,0001	<,0001	0,8817	0,3733	0,0951	0,7413	0,7799	0,5432	0,0004	0,8656	0,2076	0,9540	0,8046	0,3403	<,0001
RR tri index rs	<,0001	<,0001	<,0001	<,0001	0,0140	0,0005	<,0001	<,0001	<,0001	<,0001	0,0002	<,0001	<,0001	<,0001	<,0001	0,0136	0,2633	0,0063	0,7801	0,7201	0,9792	0,0002	0,9242	0,2699	0,5316	0,4208	0,9758	<,0001
TINN rs	0,2902	0,0002	<,0001	0,0089	0,5983	0,0030	<,0001	0,0272	0,0424	<,0001	0,2090	<,0001	0,2640	0,8817	0,0136	<,0001	0,2502	0,6996	0,6440	0,3026	0,2191	0,2478	0,3512	0,7348	0,4226	0,3761	0,7555	<,0001
Illness duration	0,7572	0,0493	0,0107	0,7846	0,9389	0,0050	0,1266	0,6509	0,6525	0,0483	0,7292	0,0050	0,1758	0,3733	0,2633	0,2502	<,0001	0,5937	0,9887	0,2284	0,2644	0,2327	0,2647	0,4103	0,2790	0,1189	0,1168	0,6067
SAS	0,0619	0,0172	0,1023	0,0244	0,8441	0,3686	0,0122	0,0324	0,7512	0,0584	0,6736	0,3884	0,1010	0,0951	0,0063	0,6996	0,5937	<,0001	<,0001	0,1067	0,1205	0,0315	0,1090	0,6787	<,0001	0,6878	0,0331	0,8491
SDS	0,7521	0,6340	0,6658	0,9827	0,8692	0,2484	0,7675	0,8170	0,1330	0,7810	0,0516	0,8804	0,9337	0,7413	0,7801	0,6440	0,9887	<,0001	<,0001	0,0007	0,4108	0,0003	0,1840	0,0011	<,0001	0,3075	0,0038	0,2408
MAF	0,8759	0,3955	0,5857	0,5966	0,3116	0,8829	0,8116	0,4718	0,3256	0,6356	0,2065	0,9988	0,6704	0,7799	0,7201	0,3026	0,2284	0,1067	0,0007	<,0001	0,3490	0,0002	0,0105	0,0002	0,0132	0,2538	0,1230	0,0556
MOS	0,7828	0,5434	0,9846	0,9959	0,8507	0,3280	0,4963	0,7815	0,9113	0,5925	0,7148	0,5097	0,7793	0,5432	0,9792	0,2191	0,2644	0,1205	0,4108	0,3490	<,0001	0,0321	0,0474	0,1940	0,7049	0,2422	0,6426	0,3989
FIQ	0,2999	0,0253	0,0044	0,2019	0,8904	0,0066	0,0015	<,0001	0,0002	0,0011	0,0004	0,0030	0,0002	0,0004	0,0002	0,2478	0,2327	0,0315	0,0003	0,0002	0,0321	<,0001	<,0001	<,0001	0,0026	0,3952	0,6777	<,0001
NRS	0,8350	0,6605	0,6425	0,9741	0,9174	0,9579	0,9338	0,6783	0,0536	0,8360	0,0532	0,5013	0,9285	0,8656	0,9242	0,3512	0,2647	0,1090	0,1840	0,0105	0,0474	<,0001	<,0001	0,0413	0,6652	0,5838	0,5932	0,0385
SF-36_PHI	0,8340	0,6077	0,3617	0,5537	0,1839	0,1140	0,3789	0,2151	0,0126	0,5836	0,0144	0,5069	0,2062	0,2076	0,2699	0,7348	0,4103	0,6787	0,0011	0,0002	0,1940	<,0001	0,0413	<,0001	0,0657	0,4845	0,3937	0,2162
SF-36_MHI	0,3603	0,2534	0,1223	0,2155	0,4291	0,7729	0,6030	0,8482	0,1844	0,6873	0,1189	0,3760	0,7803	0,9540	0,5316	0,4226	0,2790	<,0001	<,0001	0,0132	0,7049	0,0026	0,6652	0,0657	<,0001	0,6508	0,0762	0,6721
VPI	0,0352	0,2059	0,6613	0,0774	0,0018	0,6616	0,1770	0,7781	0,3375	0,2953	0,5567	0,5614	0,9503	0,8046	0,4208	0,3761	0,1189	0,6878	0,3075	0,2538	0,2422	0,3952	0,5838	0,4845	0,6508	<,0001	<,0001	0,2799
SS	0,3392	0,7520	0,0916	0,8407	0,0017	0,3716	0,7959	0,7891	0,3653	0,5153	0,7607	0,2257	0,3708	0,3403	0,9758	0,7555	0,1168	0,0331	0,0038	0,1230	0,6426	0,6777	0,5932	0,3937	0,0762	<,0001	<,0001	0,7147
HRV grade	<,0001	<,0001	<,0001	<,0001	0,1375	<,0001	<,0001	<,0001	0,0002	<,0001	0,0002	<,0001	<,0001	<,0001	<,0001	<,0001	0,6067	0,8491	0,2408	0,0556	0,3989	<,0001	0,0385	0,2162	0,6721	0,2799	0,7147	<,0001
